# ^18^F-FDG-PET-based Radiomics signature predicts MGMT promoter methylation status in primary diffuse glioma

**DOI:** 10.1186/s40644-019-0246-0

**Published:** 2019-08-19

**Authors:** Ziren Kong, Yusong Lin, Chendan Jiang, Longfei Li, Zehua Liu, Yuekun Wang, Congxin Dai, Delin Liu, Xuying Qin, Yu Wang, Zhenyu Liu, Xin Cheng, Jie Tian, Wenbin Ma

**Affiliations:** 1 Department of Neurosurgery, Peking Union Medical College Hospital, Chinese Academy of Medical Sciences and Peking Union Medical College, No.1 Shuaifuyuan Wangfujing Dongcheng District, Beijing, China; 2 Department of Nuclear Medicine, Peking Union Medical College Hospital, Chinese Academy of Medical Sciences and Peking Union Medical College, No.1 Shuaifuyuan Wangfujing Dongcheng District, Beijing, China; 3 Collaborative Innovation Center for Internet Healthcare, Zhengzhou University, No.75 Daxue Road, Erqi District, Zhengzhou, Henan China; 4 Key Laboratory of Molecular Imaging, Institute of Automation, Chinese Academy of Science, No.80 East Zhongguancun Road, Haidian District, Beijing, China; 5 Department of Pathology, Peking Union Medical College Hospital, Chinese Academy of Medical Sciences and Peking Union Medical College, No.1 Shuaifuyuan Wangfujing Dongcheng District, Beijing, China; 6 Beijing Advanced Innovation Center for Big Data-Based Precision Medicine, School of Medicine, Beihang University, No. 37 Xueyuan Road, Haidian District, Beijing, China; 7 Engineering Research Center of Molecular and Neuro Imaging of Ministry of Education, School of Life Science and Technology, Xidian University, 266 Xinglong Section of Xifeng Road, Xi’an, Shaanxi China; 8 University of Chinese Academy of Sciences, No.80 East Zhongguancun Road, Haidian District, Beijing, China; 9 Key Laboratory of Industrial Microbiology, Tianjin University of Science and Technology, No. 1038 Dagu Nanlu, Hexi District, Tianjin, China; 10 School of Software, Zhengzhou University, No.75 Daxue Road, Erqi District, Zhengzhou, Henan China

**Keywords:** Radiomics, FDG PET, MGMT promoter methylation, Glioma, Prognosis

## Abstract

**Background:**

The methylation status of the O^6^-methylguanine-DNA methyltransferase (MGMT) promoter has emerged as a favorable independent prognostic and predictive biomarker in glioma. This study aimed to build a radiomics signature based on ^18^F-fluorodeoxyglucose (FDG) positron emission tomography (PET) for noninvasive measurement of the MGMT promoter methylation status in glioma.

**Methods:**

One hundred and seven pathology-confirmed primary diffuse glioma patients were retrospectively included and randomly assigned to the primary (*n* = 71) or validation cohort (*n* = 36). The MGMT promoter methylation status was measured by pyrosequencing. A total of 1561 radiomics features were extracted from the three-dimensional region of interest (ROI) on the standard uptake value (SUV) maps that were generated from the original ^18^F-FDG PET data. A radiomics signature, a clinical signature and a fusion signature that combined the clinical and radiomics features together were generated. The performance of the three signatures was evaluated by receiver operating characteristic (ROC) curve analysis, and the patient prognosis was stratified based on the MGMT promoter methylation status and the signature with the best performance.

**Results:**

Five radiomics features were selected to construct the radiomics signature, and displayed the best performance with area under the receiver operating characteristic (ROC) curve (AUC) reaching 0.94 and 0.86 in the primary and validation cohorts, respectively, which outweigh the performances of clinical signature and fusion signature. With a median follow-up time of 32.4 months, the radiomics signature stratified the glioma patients into two risk groups with significantly different prognoses (*p* = 0.04).

**Conclusions:**

^18^F-FDG-PET-based radiomics is a promising approach for preoperatively evaluating the MGMT promoter methylation status in glioma and predicting the prognosis of glioma patients noninvasively.

## Background

Glioma is one of the most malignant central nervous system (CNS) tumors, with an annual incidence of 5.26 per 100,000 individuals [1]. Alkylating agents, such as temozolomide (TMZ), induce guanine-alkyl groups to the DNA and trigger tumor cell death, and have been widely utilized in the treatment of glioma [2, 3]. This methylation damage to DNA can be remedied by a DNA repair enzyme, O^6^-methylguanine-DNA methyltransferase (MGMT), which can be epigenetically silenced according to its promoter methylation status, making the MGMT promoter methylation status a strong prognostic and predictive biomarker in glioma [3–5] that is routinely measured in the clinical evaluation of glioma patients. However, the MGMT status is mainly assessed based on tumor samples by pyrosequencing, methylation-specific polymerase chain reaction (PCR) or methylation chip analysis [6–8], and these methods are restricted by comparatively long detection periods and high detection costs, the existence of intratumor heterogeneity, and the unattainability of tumor samples through surgery or biopsy. Therefore, noninvasive measurement of the MGMT promoter methylation status has great clinical significance to precisely guide treatment and predict prognosis.

Radiomics, a recently emerging technique for quantifying tumor characteristics with high-throughput radiomics features, allows prediction of the tumor phenotype through mathematic models that are built with selected radiomics features [9]. Current radiomics studies in the glioma field have shown promising results in demonstrating correlations between magnetic resonance imaging (MRI) features and clinical manifestations [10], WHO grades [11], molecular characteristics [12–15], and prognoses [16]. Specifically, Li et al. and Xi et al. predicted the MGMT promoter methylation status in glioblastoma [13, 14] and Wei et al. investigated the imaging features of WHO grade II-IV astrocytoma [15] using radiomics, suggesting the efficacy of using radiomics to predict the MGMT promoter methylation status.

^18^F-fluorodeoxyglucose (FDG) positron emission tomography (PET) is an alternative molecular imaging technique that has been applied to tumor grading [17], surgical planning [18], recurrence identification [19], and prognosis prediction [20] in glioma. In particular, Choi et al. found that MGMT-methylated WHO grade III and IV gliomas had a significantly higher maximum tumor-to-normal tissue uptake ratio (TNR) and identified a trend of higher mean TNRs in MGMT-methylated gliomas than in MGMT-unmethylated gliomas [21]. In addition, Colavolpe et al. reported a case of multicentric glioblastoma in which the lesion showed higher MGMT expression and intense ^18^F-FDG uptake [22], suggesting a potential correlation between the ^18^F-FDG-PET results and the MGMT promoter methylation status in glioma. However, to the best of our knowledge, no studies have focused on predicting the MGMT promoter methylation status using an ^18^F-FDG-PET-based radiomics approach. Since the MGMT promoter methylation status has been proven to be an independent prognostic and predictive marker in glioma regardless of the WHO classification or chemotherapy regimen [3–5, 23, 24], prediction of the MGMT promoter methylation status using ^18^F-FDG-PET radiomics may have great clinical potential.

This study retrospectively investigated the radiomics characteristics of gliomas by ^18^F-FDG-PET to build a conceivable model for predicting the MGMT promoter methylation status and patient prognosis noninvasively.

## Methods

### Patients

**Fig. 1:**
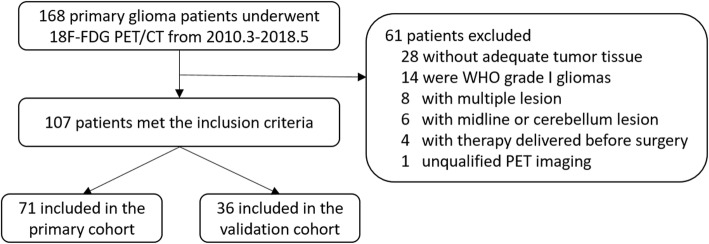
Patient recruitment pathway. A total of 168 patients were screened, and 107 patients were included in the current study. Patients were randomly assigned to the primary or validation cohort

Patients who were pathologically diagnosed with primary glioma and underwent an ^18^F-FDG-PET/CT examination between March 2010 and May 2018 at Peking Union Medical College Hospital were retrospectively reviewed. The inclusion criteria included the following: 1) adults with histopathologically confirmed WHO grade II-IV primary diffuse glioma without a previous history of CNS tumors; 2) preoperative ^18^F-FDG PET/CT examination of the brain; 3) sufficient paraffin-embedded tumor tissue for measurement of the MGMT promoter methylation status; and 4) no chemotherapy or radiotherapy delivered before ^18^F-FDG PET/CT acquisition and surgery. The study design was approved by the Institutional Review Board, and all patients provided informed consent. A total of 107 patients met the inclusion criteria and were randomly assigned to the primary cohort (*n* = 71) or the validation cohort (*n* = 36). The patient recruitment pathway is displayed in Fig. 1.

### MGMT promoter methylation status measurement

The methylation status of the MGMT promoter was measured by pyrosequencing, as previously described [25]. Briefly, DNA was extracted from formalin-fixed, paraffin-embedded tumor samples with a Simplex OUP® FFPE DNA Extraction Kit (TIB, China) and quantified by spectrophotometry with a NanoDrop 2000 system (Thermo Fisher, US). Bisulfate modification was performed with an EpiTect Bisulfite Kit (Qiagen, Germany), and PCR was carried out with a DRR007 Kit (Takara, Japan) using a Verity 96-Well Thermal Cycler (Thermo Fisher, US). Pyrosequencing was subsequently performed in 10 CpG island regions within the MGMT promoter using the PyroMark Q96 system (Qiagen, Germany). Gliomas were defined as having a methylated MGMT promoter if the average methylation rate of the CpG regions was greater than or equal to 8%; gliomas were defined as having an unmethylated MGMT promoter if the average methylation rate was less than 8% [25].

### ^18^F-FDG-PET/CT data acquisition

^18^F-FDG was produced using an RDS-111 Cyclotron (CTI, US). A dose of 5.55 MBq (0.15 mCi) ^18^F-FDG per kilogram of body weight was intravenously administered after the patient had fasted at least 4 h and their blood glucose level was determined not to exceed the normal limit (6.4 mM). The patient underwent ^18^F-FDG-PET/CT on a Biograph 64 TruePoint TrueV PET/CT system (Siemens Medical Solutions, Germany) after a 40–60 min time lag in standardized conditions (quiet, dimly lit room with patient’s eyes closed), and acquired 148 axial slides with an interslice spacing of 3 mm.

### Tumor segmentation

The three-dimensional region of interest (ROI) was segmented by two experienced neurosurgeons for the ^18^F-FDG-PET data on the merged PET/CT images using ITK-SNAP software (http://www.itksnap.org/pmwiki/pmwiki.php), with patients’ contrast-enhanced T1-weighted images (for contrast-enhanced tumors) and T2-weighted fluid attenuated inversion recovery (FLAIR) images (for non-contrast-enhanced tumors) as anatomical reference. The ROIs were subsequently reviewed by a senior nuclear medical scientist blinded to the patients’ information. If there was a discrepancy of less than 5% between the ROIs placed by the two neurosurgeons, the final ROI was defined as the region of overlap, and if the discrepancy was greater than or equal to 5%, the nuclear medical scientist made the final decision.

### Radiomics feature extraction and selection

Standard uptake value (SUV) maps were generated from the original ^18^F-FDG-PET DICOM data using MATLAB version R2015b (Math Works, US). A total of 1561 radiomics features, including 13 shape and size features, 18 first-order features, 68 texture features, 688 wavelet features and 680 further filtered (logarithm, square, exponential, gradient, square root, lbp-2D, lbp-3D) features were extracted using PyRadiomics (https://github.com/Radiomics/pyradiomics) [26]. The radiomics features were normalized to the interval of 0 to 1.

The radiomics features were reduced and selected through sequential application of the Wilcoxon rank-sum test and multivariate linear logistic regression with the L1 penalty.

### Clinical feature evaluation

Five clinical features, respectively, age, sex, metabolic pattern (cystic or solid), SUVmax and SUVmean, were also evaluated. Cystic metabolic tumor was defined as a lesion with visible marginal ^18^F-FDG update but significant low central radioactivity, and solid metabolic tumor was defined as a lesion without a significant low metabolic necrosis or cysts inside the ROI [27, 28]. SUVmax and SUVmean were defined as radiomics feature ‘First order_Maximum’ and ‘First order_Mean’ that extracted from the ROI.

### Signature construction, validation and evaluation

Three predictive signatures, namely, a radiomics signature, clinical signature, and fusion signature, were constructed. The radiomics signature was generated with the radiomics features that were previously selected with a support vector machine (SVM). The clinical signature was generated with 5 clinical features using the logistic regression after selection by the Akaike information criterion (AIC). The selected clinical features and selected radiomics features were combined to generate the fusion signature using the logistic regression. The 3 signatures were independently validated in the validation cohort.

The signatures were evaluated in terms of the area under the receiver operating characteristic (ROC) curve (AUC), accuracy, sensitivity, specificity, and positive and negative predictive values. Decision curve analysis was applied to reflect the clinical utility of the model [29, 30], and the Delong test was utilized to evaluate the difference in the performance of the models.

### Prognosis analysis

The overall survival (OS) of patients was evaluated up to May 31, 2018. Kaplan-Meier curves were plotted based on the MGMT promoter methylation status and the signature with the best performance in stratifying the OS of patients. The log-rank test was utilized to determine differences in survival between the groups.

### Statistical analysis

Statistical analysis was performed with SPSS Statistics software, version 18.0 (Chicago, US) and R software, version 3.4.1 (https://www.r-project.org/). Statistically significant differences were defined by a two-tailed threshold of *p* < 0.05.

## Results

### Clinical characteristics

The clinical characteristics of patients in the primary and validation cohorts are summarized in Table 1. The MGMT methylation rate in the primary and validation cohorts was 54.9 and 55.6%, respectively. There were no significant interclass differences in age, sex, body weight, metabolic pattern, WHO grade, SUVmax or SUVmean among the included patients (*p* = 0.11–0.84). However, tumors with MGMT promoter methylation tend to have a higher rate for cystic metabolic pattern, and the difference of metabolic pattern for MGMT methylated and MGMT unmethylated patients reached statistical significance in the validation cohort (*p* = 0.20 and 0.02 in the primary and validation cohort, respectively).

**Table 1:** Patients’ Characteristics of Primary and Validation Cohorts

Characteristics	Primary cohort (*n* = 71)			Validation cohort (*n* = 36)			*P*
	Methylated (*n* = 39)	Unmethylated (*n* = 32)	*P*	Methylated (*n* = 20)	Unmethylated (*n* = 16)	*P*	
Age (mean ± SD, years)	50.72 ± 14.01	50.50 ± 14.82	0.95	46.70 ± 12.45	58.33 ± 11.95	0.08	0.65
Gender			0.97			0.45	0.84
Male	23	19		10	10		
Female	16	13		10	6		
Weight (mean ± SD, kg)	67.24 ± 12.36	64.20 ± 10.11	0.27	69.05 ± 14.74	66.28 ± 9.56	0.50	0.45
Metabolic Pattern			0.20			0.02	0.34
Cystic	23	14		15	6		
Solid	16	18		5	10		
WHO Grading			0.05			0.08	0.11
Low Grade Glioma	13	2		11	2		
High Grade Glioma	26	30		9	14		
SUVmax	9.18 ± 4.05	10.51 ± 4.45	0.20	9.89 ± 4.11	7.84 ± 3.28	0.11	0.33
SUVmean	4.00 ± 2.10	4.60 ± 1.86	0.22	4.31 ± 2.01	3.40 ± 1.59	0.14	0.36

*Abbreviations: SD* Standard deviation, *WHO* World Health Organization, *SUV* Standard uptake value

*Note*: Chi-Square or Fisher Exact tests, as appropriate, were used to compare the differences in categorical variables, while the independent sample *t*-test was used to compare the differences in age

### Feature selection and signature construction

Among the 1561 extracted radiomics features, 1543 redundant features were reduced through the Wilcoxon rank-sum test, and 5 final features were selected by logistic regression with the L1 penalty to build the radiomics signature. Only the metabolic pattern was selected by the AIC to build the clinical signature, and the fusion signature was built based on the radiomics signature and metabolic pattern. The selected radiomics features are shown in Table 2.

**Table 2:** Selected Features in the Radiomics Signature

Feature Name	Matrix	Filter
Skewness	First Order	Logarithm
90 Percentile	First Order	Logarithm
Median	First Order	Logarithm
Joint Average	GLCM	Logarithm
Maximum 2D Diameter Slice	Shape	Original

*Abbreviations*: GLCM, Gray-Level Co-occurrence Matrix; 2D, two-dimensional

### Diagnostic performance of the three signatures

**Fig. 2:**
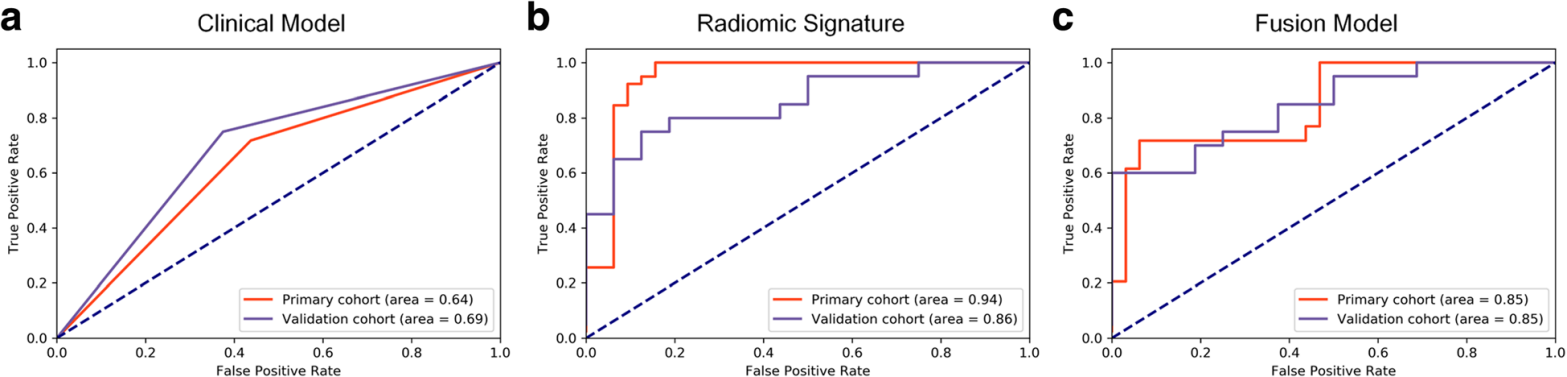
Receiver operating characteristic (ROC) curves of the prediction models. ROC curve of the clinical (**a**), radiomics (**b**), and fusion (**c**) predictive models in both the primary and validation cohorts

**Fig. 3:**
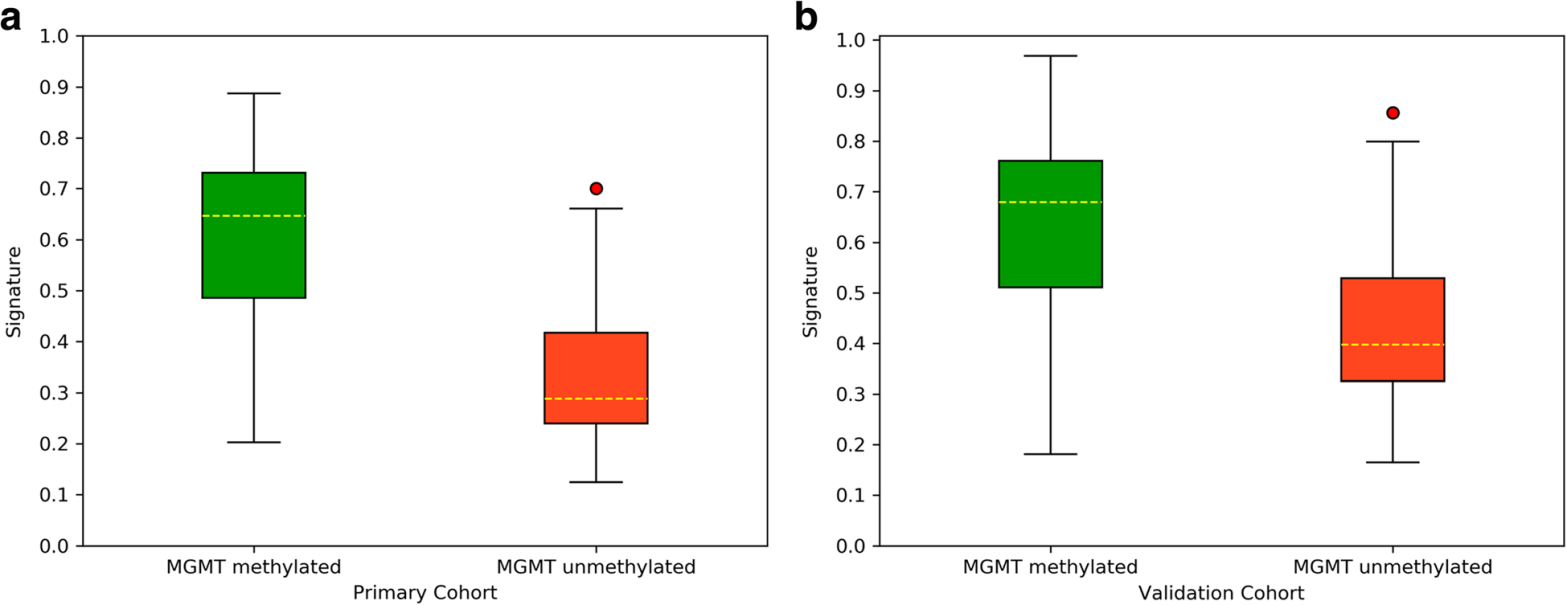
Box plots of the radiomics signature. Box plots of the radiomics signature in the primary (**a**) and validation cohorts (**b**). The signature displayed a higher value for the patients with MGMT-methylated tumors in both cohorts

**Fig. 4:**
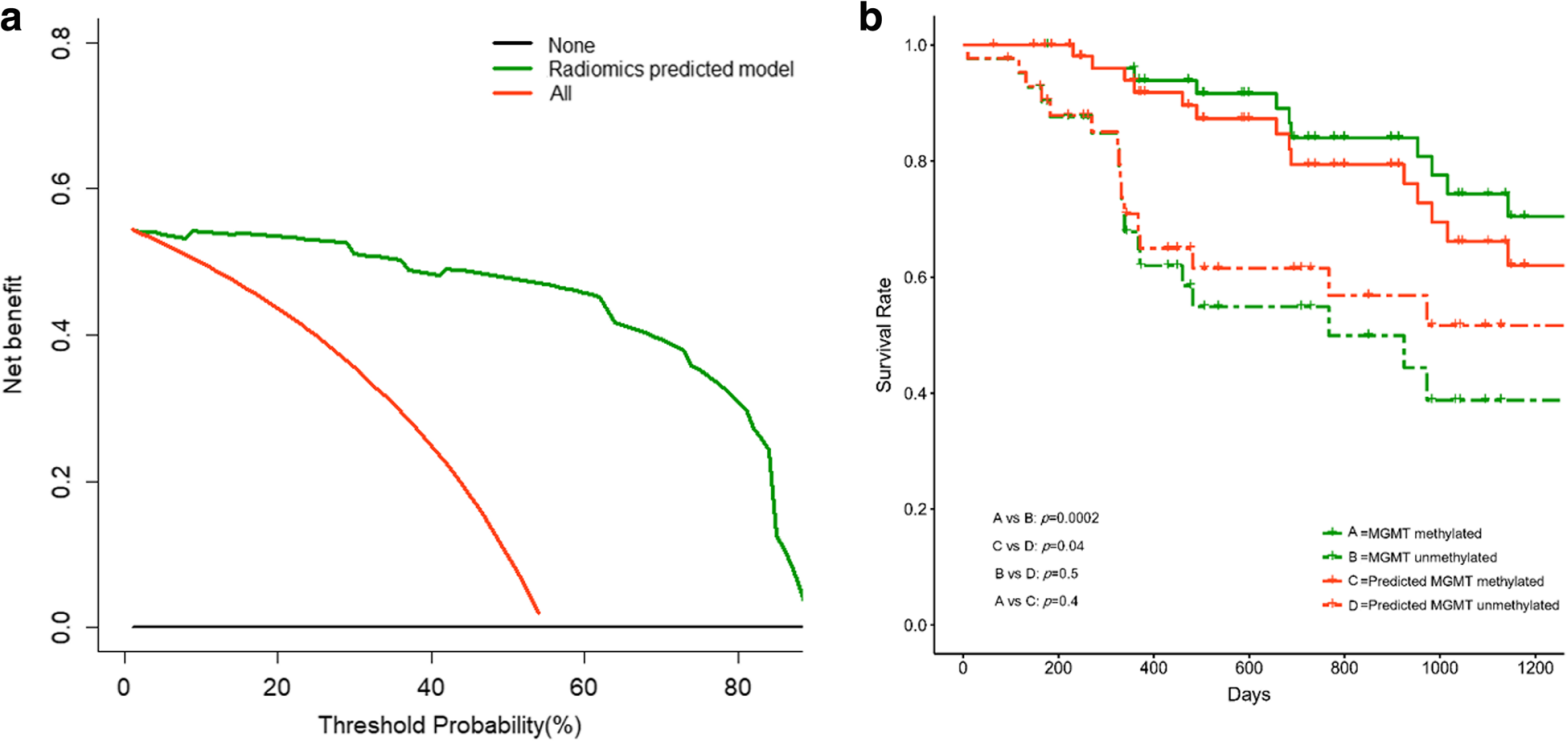
Clinical utility of the radiomics signature. The decision curve of the radiomics signature in the primary cohort (**a**). The x axis represented the threshold probability, where the expected benefit of treatment as MGMT methylated is equal to the expected benefit of treatment as MGMT unmethylated (the threshold probability varies from patient to patient). The y axis indicated the net benefit for the treatment which considered the benefit of true positive and loss of false positive, and higher net benefit value indicates better model. The net benefit of the radiomics signature is further compared with the default strategies, which we treat all patients as MGMT methylated (red line) or as MGMT unmethylated (black line). The current prediction model outweigh both default strategies at any threshold probability, suggesting the clinical value of our model at all circumstances. Kaplan-Meier curves revealed the prognosis-based groups stratified by the MGMT promoter methylation status and the radiomics signature (**b**)

The radiomics signature performed the best among the three signatures in predicting the MGMT promoter methylation status, reaching an AUC of 0.94 in the primary cohort and 0.86 in the validation cohort. The clinical signature demonstrated a moderate predictive value and reached an AUC of 0.64 and 0.69 in the primary and validation cohorts, respectively. The fusion signature performed better than the clinical signature but poorer than the radiomics signature, with an AUC of 0.85 both in the primary and validation cohorts. The Delong test demonstrated that the radiomics signature performed significantly better than the clinical and fusion signatures in the primary cohort (*p* < 0.0001 and *p* = 0.036, respectively), but the differences in the validation cohort were not significant (*p* = 0.115 and 0.900, respectively) due to the limited number of patients. The decision curve reflecting the benefit of the radiomics signature showed a net benefit outweighing both schemes at any threshold probability in the primary cohort. The performance of the radiomics, clinical and fusion signatures is summarized in Table 3. The ROC curves are displayed in Fig. 2, and the box plots are demonstrated in Fig. 3. The decision curve is shown in Fig. 4 (a).

**Table 3:** The Performances of the Three Predictive Models

Models	AUC (95%CI)	ACC (95%CI)	SEN (95%CI)	SPE (95%CI)	PPV (95%CI)	NPV (95% CI)
Radiomics model						
Primary cohort	0.94 (0.93, 0.96)	91.3% (89.8, 93.3)	94.9% (93.1, 96.6)	87.5% (84.4, 90.7)	90.2% (87.8, 92.8)	93.3% (91.1, 95.6)
Validation cohort	0.86 (0.83, 0.88)	77.8% (75.2, 80.3)	75.0% (71.5, 78.6)	81.3% (77.5, 84.9)	83.3% (80.0, 86.7)	72.2% (68.2, 76.1)
Clinical model						
Primary cohort	0.64 (0.61, 0.67)	64.8% (61.9, 67.9)	71.8% (68.1, 75.7)	56.3% (51.6, 61.1)	66.7% (62.9, 70.6)	62.1% (57.3, 67.2)
Validation cohort	0.69 (0.66, 0.72)	66.4% (66.6, 72.3)	75.0% (71.3, 78.5)	62.5% (58.1, 67.0)	71.4% (67.9, 75.1)	66.7% (61.9, 71.1)
Fusion model						
Primary cohort	0.85 (0.83, 0.87)	64.8% (62.0, 67.7)	71.8% (68.1, 75.5)	56.3% (51.6, 60.9)	66.7% (63.0, 70.4)	62.1% (57.5, 66.7)
Validation cohort	0.85 (0.82, 0.87)	72.7% (78.1, 82.9)	80.0% (76.6, 83.5)	62.5% (58.0, 67.0)	72.7% (69.3, 76.3)	71.4% (66.7, 76.1)

*Abbreviations: CI* Confidence interval, *AUC* Area under receiver-operating characteristic curve, *ACC* Accuracy, *SEN* Sensitivity, *SPE* Specificity, *PPV* Positive predictive value, *NPV* Negative predictive value

### Prognostic performance of the Radiomics signature

Among the 107 included patients, 100 patients who were known to survive to the closing date or to have an exact time of death were included in the prognosis analysis, and the median follow-up time is 32.4 months. Both the MGMT promoter methylation status and the radiomics signature stratified the glioma patients into a high-risk group and a low-risk group (*p* = 0.0002 and 0.04, respectively), and the differences within the high- and low-risk groups did not reach statistical significance. The Kaplan-Meier curves are shown in Fig. 4 (b).

## Discussion

In this study, ^18^F-FDG-PET radiomics features were extracted, selected and analyzed, and three prediction signatures, respectively, and a radiomics signature, a clinical signature, and a fusion signature, were built to predict the MGMT promoter methylation status. The radiomics signature displayed the best performance, with an accuracy of 91.3% and an AUC of 0.94 in the primary cohort, and an accuracy of 77.8% and an AUC of 0.86 in the validation cohort, respectively. The clinical value of the radiomics signature was further demonstrated by the prognosis analysis. These results suggest that ^18^F-FDG-PET-based radiomics is a promising method for predicting the MGMT promoter methylation status and prognosis noninvasively, demonstrating strong potential for clinical application.

Previous studies on radiological evaluation of the MGMT promoter methylation status have mainly focused on the visual features, quantitative parameters or high-throughput radiomics features [13–15, 31–34] of gliomas (mostly glioblastomas) based on multimodal MRI and have reported accuracies ranging from 0.58–0.89 and AUCs ranging from 0.75–0.92 (without distinguishing training and validation data). Our prediction model demonstrated comparable accuracy and AUC values, suggesting the capability of ^18^F-FDG-PET radiomics to predict the MGMT promoter methylation status. However, most previous studies on imaging-based prediction of the MGMT promoter methylation status have mainly focused on glioblastomas, and limited studies have included less aggressive gliomas (e.g., lower grade gliomas, such as WHO grade II and III gliomas), in which the MGMT promoter status also has prognostic and predictive value [3–5, 23, 24]. Although there may be discriminative imaging characteristics, our ^18^F-FDG-PET-based radiomics signature can predict the MGMT promoter methylation status regardless of the WHO grade (e.g., in lower grade gliomas and glioblastomas) or pathological information (e.g., in astrocytomas and oligodendrogliomas), suggesting the capability of noninvasive prediction without previous knowledge based on tumor samples.

Unlike MRI, which displays the structural characteristics of tumors, PET is a highly sensitive molecular imaging technique that reflects the altered tumor metabolism that is ubiquitous among cancer cells. Malignant brain tumors usually exhibit an altered glucose metabolism, in which glucose is converted to pyruvate and further into lactate instead of entering mitochondria and the citric acid cycle [35]. ^18^F-FDG, a glucose analogue, can be taken up by cells but not further catabolized through glycolysis, making it a reliable radiotracer for measuring cancer cell metabolism. Considering the relationship between the glucose metabolism and oncogenic reprogramming [36], radiogenomic analysis based on ^18^F-FDG-PET may reflect certain molecular processes through imaging data, which is the theoretical basis of our study. However, compared with anatomical imaging modalities (e.g., CT and MRI), ^18^F-FDG-PET has a relatively low spatial resolution, which limits the stability and accuracy of certain features, especially in lesions with a relatively small volume [37].

**Fig. 5:**
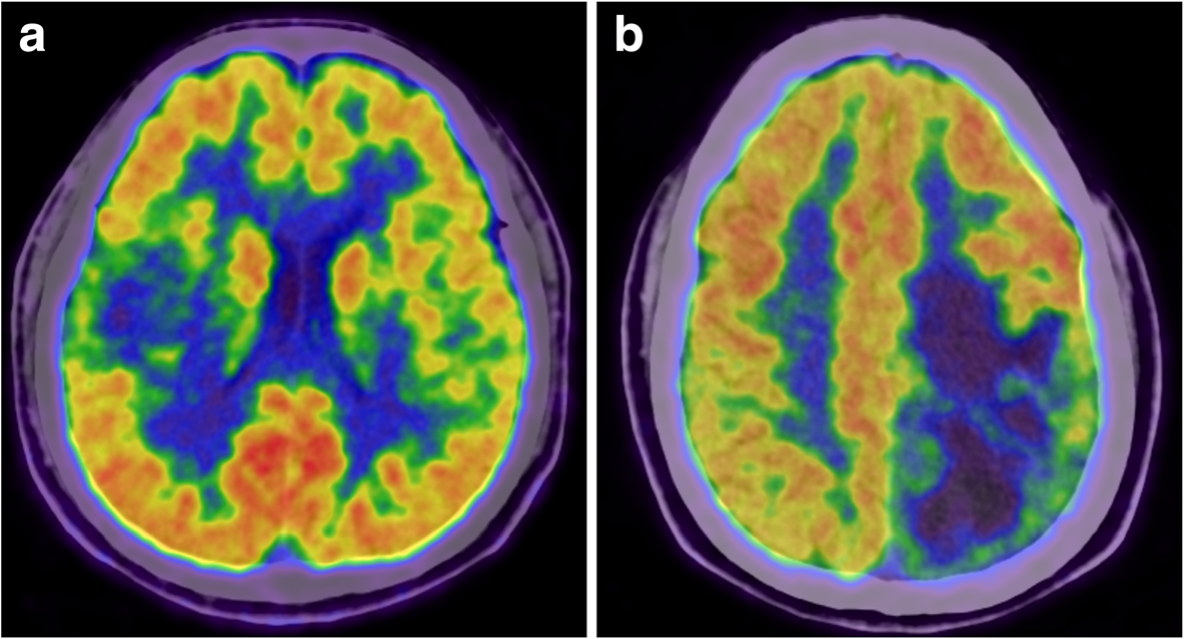
Examples of using the radiomics signature to evaluate the MGMT promoter methylation status noninvasively. A 37/M was histopathologically diagnosed with anaplastic astrocytoma with a methylated MGMT promoter (**a**), and a 44/M was histopathologically diagnosed with anaplastic astrocytoma with an unmethylated MGMT promoter (**b**). Determination of the MGMT promoter methylation status is difficult based on clinical and visually assessed imaging characteristics, but the radiomics signature demonstrated values of 0.84 (**a**) and 0.27 (**b**) in these two patients and successfully predicted their MGMT status (the cutoff value of the radiomics signature was 0.50)

Feature selection is a core step in radiomics studies since most features have little relevance to the MGMT promoter methylation status and may overwhelm the distinguishable features if they cannot be effectively reduced. The number of final selected features also needs to be balanced according to the patient cohort size because the addition of relevant features may increase performance in the primary cohort but may also result in overfitting of the radiomics signature. In our study, the Wilcoxon rank-sum test removed 1543 of the 1561 radiomics features that were irrelevant to the MGMT promoter methylation status, and logistic regression with the L1 penalty diluted the weights, allowing selection of the final 5 radiomics features to construct the radiomics signature. Although the selected radiomics features are not visually available to nuclear medicine physicians (though they are mathematically easy to comprehend), the radiomics signature did provide additional assistance to physicians in the noninvasive molecular diagnosis of glioma (Fig. 5).

Three signatures were built in our study to predict the MGMT promoter methylation status. In addition to the radiomics signature, the clinical signature was built with visualized imaging features (e.g., metabolic pattern), and the fusion signature was built with the 5 selected radiomics features and the metabolic pattern. However, the radiomics signature demonstrated the best performance and outweighed the clinical signature in both the primary and validation cohorts, suggesting that the selected radiomics features are more reliable than the clinically assessed imaging features in differentiating tumors based on the MGMT promotor methylation status. Objective clinical features (e.g., age and sex) and the most frequently used quantitative imaging parameters (e.g., SUVmax and SUVmean) were excluded by the AIC when building the clinical signature, although some of these features are the only references for physicians in noninvasively evaluating the MGMT promoter methylation status without radiomics. Moreover, the addition of the clinical feature (i.e., metabolic pattern) to the set of radiomics features decreased the AUC of the prediction model, indicating a potential disturbance to the signature with the addition of features with less relevance. Thus, clinical features may not be integrated into the noninvasive radiomics evaluation of the MGMT promoter methylation status.

The MGMT promoter has proven to be a strong prognostic biomarker in glioma. The retrospective investigation of the EORTC 26981/22981 trial demonstrated that the MGMT promoter methylation status is a favorable independent prognostic biomarker in glioblastoma [5, 6]; the NOA-04 trial and the EORTC 26951/26053/22054 trial demonstrated its prognostic value in anaplastic glioma regardless of the histopathological classification and treatment strategy [3, 23, 24]. The recently reported RTOG 0424 trial also suggests that the MGMT promotor methylation status can predict the prognosis of patients with low-grade glioma treated with radiotherapy and TMZ [4]. In accordance with previous evidences, patients with MGMT promoter methylation displayed significantly longer OS in our research. The clinical use of a radiomics signature can be further supported if the signature not only detects the MGMT promotor methylation status noninvasively but also predicts the patients’ prognosis before treatment. In our study, the radiomics signature could stratify patients into two significantly different groups based on the prognosis, suggesting the feasibility of using the radiomics signature to predict prognosis in addition to distinguishing molecular features. Moreover, the differences between the MGMT promotor methylation status-predicted and radiomics signature-predicted prognosis within each risk group were nonsignificant, even with population discrepancies within each risk group (e.g., a 20% difference in the composition of the low-risk group), indicating that the radiomics signature can serve to evaluate the prognosis aside from the MGMT promoter methylation status. Despite the results from the EORTC 26981/22981/26053/22054 and NOA-04 trials suggesting that the MGMT promoter methylation status is a predictive biomarker that can be used to evaluate whether a patient will benefit from TMZ [3, 5, 6, 24], chemotherapy strategies were not integrated into the prognosis analysis due to their diversity and the retrospective nature of this study.

The current study has several limitations. First, this was a single-center, retrospective study with a limited sample size, and the validation cohort is particularly restricted. Further prospective, multicenter studies with large patient cohorts may be essential for improving the generality and performance of the prediction model. Second, there may be a selection bias of the included patients since ^18^F-FDG-PET examination was not mandatorily performed. The necessity of differential diagnosis of the intracranial lesion or the evaluation of the extracranial situation were the major consideration to suggest an ^18^F-FDG-PET scan. Third, the radiomics model was constructed without subclassification of the metabolic pattern (i.e., solid or cystic) and therefore may not include distinguishable features for determining the MGMT promoter methylation status in each subclassification. Fourth, more than half of the patients did not reach the endpoint of the prognosis analysis, which may have introduced bias to the prognosis data. Further studies with long-term follow-up periods may be needed to eliminate such imbalances. Finally, in addition to ^18^F-FDG-PET data, multimodality imaging data (e.g., data from MRI and PET with alternative tracers) may be further integrated into the radiomics model for predicting the MGMT promoter methylation status in glioma.

## Conclusions

^18^F-FDG-PET-based radiomics is a promising method for preoperatively evaluating the MGMT promoter methylation status in glioma and has the potential to guide the treatment and predict the prognosis of glioma patients noninvasively.

## Data Availability

The datasets used and analyzed in the current study are available from the corresponding author on reasonable request.

## Abbreviations

AIC: Akaike information criterion
AUC: Area under the ROC curve
CNS: Central nervous system
FDG: Fluorodeoxyglucose
MGMT: O^6^-methylguanine-DNA methyltransferase
MRI: Magnetic resonance imaging
OS: overall survival
PCR: Polymerase chain reaction
PET: Positron emission tomography
RFE: Recursive feature elimination
ROC: Receiver operating characteristic
ROI: Region of interest
SVM: Support vector machine
TMZ: Temozolomide
TNR: Tumor-to-normal tissue uptake ratio
